# Impact of cysteine mutations on the structural dynamics and functional impairment of SOD1: insights into the pathogenicity of amyotrophic lateral sclerosis

**DOI:** 10.1186/s44342-025-00041-8

**Published:** 2025-03-06

**Authors:** Jessica Jeejan, Lawanya Rao, Shivank Sadasivan, Richa Lopes, Norine Dsouza

**Affiliations:** Department of Biotechnology, St. Xavier’s College, Maharashtra Mumbai, India

**Keywords:** ALS, Missense mutations, Neurodegenerative, SOD1, Dimerisation

## Abstract

Amyotrophic lateral sclerosis (ALS) is a rare neurodegenerative disease prevalent in American and European populations, with its onset and progression significantly influenced by mutations in the superoxide dismutase 1 (SOD1) protein. While previous studies have highlighted the effects of mutations in the metal-binding region and catalytic region and dimerisation of SOD1, the impact of mutations involving the *Cysteine* residue at the N-terminal end remains unexplored. This study investigates the effects of *Cysteine*-to-*Trp, Phe, Ser,* and *Gly* mutations at the 6th position of SOD1’s N-terminal end on its structural dynamics and functional impairment. Our computational analysis using PolyPhen-2, PROVEAN, Meta-SNP, and PhD-SNP predicted mutations to be deleterious, with their negative impacts likely contributing to disease development. Furthermore, stability studies and bonding pattern changes due to the mutations, analysed by mCSM, SDM, DUET, Dynamut2, and PremPS revealed changes in free energy and disruption in intramolecular interactions. The molecular dynamics studies revealed distinct changes in stability patterns among the mutations, particularly in *Cys6Trp* and *Cys6Phe*. All the mutations primarily altered the catalytic region of the protein; additionally, *Cys6Phe* and *Cys6Gly* caused disruption in the metal-binding region. The impact of mutations on the dimerisation of SOD1, analysed using MM/PBSA showed destabilisation due to *Cys6Phe *mutation. These findings provide molecular insights into the clinical symptoms observed in patients, highlighting the critical impact of the *Cys6Phe* mutation on the metal-binding and catalytic loops of SOD1 along with destabilisation of dimer formation. Overall, our analysis offers valuable insights into the molecular mechanisms driving structural changes in SOD1 due to mutations, contributing to a deeper understanding of their role in ALS pathogenicity.

## Introduction

Superoxide dismutases (SOD) are a group of enzymes that play a crucial role in converting superoxide radicals into oxygen and hydrogen peroxide. The catalytic activity of SOD relies on the association of metal binding loop with specific metal ions, such as manganese, iron, nickel, and copper, alongside electrostatic loop regions [1]. Based on the cellular location and complex formation with the metal ions, three different SODs have been found in humans: cytoplasmic SOD1 (Cu/ZnSOD1), mitochondrial SOD2 (MnSOD2), and extracellular SOD3 (Cu/ZnSOD), which play a crucial role in mitigating cellular oxidative stress [2]. Among the different types of SODs, cytosolic SOD1 has been reported to play an important role in controlling signal transduction pathways that involve reactive oxygen species (ROS). In addition, SOD1 also facilitates multiple cellular defence mechanisms, such as the activation of gene transcription as a response to neurotoxic stimuli, aids in mitochondrial function and prevents the oxidative damage due to ageing [3, 4].

SOD1 plays a role in transforming harmful superoxide radicals into less toxic compounds and is gaining researcher’s attention due to its clinical significance and association with neurological conditions like Parkinson’s disease, Alzheimer’s disease, amyotrophic lateral sclerosis (ALS), chronic inflammatory diseases and several cancers [2, 5]. The association of SOD1 with ALS is well studied and this metalloprotein has been found to aggregate in individuals with ALS, playing a role in the development and progression of the disease [4]. Numerous investigations have revealed that SOD1 misfolding and aggregation play a major role in the different forms of ALS. This structural impairment has been reported in cases of sporadic ALS, and SOD1‐linked familial ALS (fALS) [6–8].

SOD1 is a 32 Kda homodimer protein consisting of 153 amino acid residues, and each subunit of the homodimer forms complexes with Zn/Cu. The monomer of SOD1 consists of eight beta sheets that form a beta-barrel fold, joined by seven loops. Among these structural loops, the electrostatic loop (Loop VII) and the metal binding loop (Loop IV) confer catalytic activity and stability to SOD1, respectively. The electrostatic loop, which ranges from 122 to 143, participates in SOD1’s catalytic activity by binding to the substrate. Meanwhile, the metal binding loop (49th–84th) is complexed with Zn/Cu [9, 10]. The metal binding loop of SOD1 is subdivided into dimer interface sub loop (region 49–54), disulphide sub loop (region 55–61) and zinc-binding sub loop (region 62–83) [11, 12]. Further, the structural integrity of the SOD1 subunit is maintained by the intra-subunit disulphide bonds occurring between the *Cys56* and* Cys146* [13]. Studies have also shown that the disruption of intramolecular disulphide bond leading to the dissociation of metal ions in SOD1 serves as the pivotal point initiating aggregation, thereby facilitating aggregate formation among ALS patients [10, 14].

In the case of SOD1, the stability of the protein relies on several key factors that counteract aggregation tendencies which include the formation of dimer. The formation and stability of a dimer are favoured by the ability to bind to the metal cofactors, post-translational modifications at *Cys6*, and extensive hydrogen bonding at the dimer interface [2, 15]. Additionally, hydrophobic amino acids like *G51*, *G114*, and *I151* present at the dimer interface play a critical role in conferring stability to the SOD1 protein [9]. Studies have reported that mutations, both near and distant from the dimer interface, impact the stability of the protein and promote the formation of monomeric SOD1 [16]. Overall, mutations in SOD1 impact the metal-binding and catalytic regions. Additionally, they have been reported to disrupt dimer stability, leading to misfolding and protein aggregation—a hallmark of fALS [16, 17].

Gene screening studies have identified several missense mutations in the SOD1 protein in individuals with ALS. These mutations are correlated with the nature of disease progression, severity, and the survival span of affected individuals [18]. Many studies to date have found an association between the missense mutations in SOD1 and protein aggregation, indicating that certain missense mutations can alter SOD1 structure. These mutations cause structural alterations, loss of metal binding ability, and a rise in beta-sheet propensity [18–21]. In addition, mutations associated with familial ALS (fALS) are present in key structural regions of SOD1, including the dimer interface, β-sheets, loop regions, disulphide bonds, and catalytic sites—specifically, the Zn/Cu binding sites [22]. SOD1 contains four cysteine residues; among them, *Cys57* and *Cys146* establish a disulphide bridge within the monomer, while *Cys6* and *Cys111* do not form a disulphide bridge. The unpaired *Cys111* is found on the surface of the protein in the vicinity of the dimer interface, while *Cys6* is found at the N-terminal end of the protein, forming a part of a tightly bound beta-sheet [23, 24]. Several studies have linked the mutations present in unpaired *Cys6* and *Cys111* with SOD1 aggregation [25, 26]. The study by Perri, E. R., et al. found that altering the residues *Cys6* and *Cys111* in SOD1 A4V mutants led to protein misfolding, which, in turn, resulted in reduced ER stress, decreased inclusion formation, and decreased apoptosis in neuronal cells [26].

To aid in research studies, missense mutations in the SOD1 gene and protein have been reported in disease-specific databases like ALSoD (Amyotrophic Lateral Sclerosis online Database), a repository of gene mutations associated with ALS patients [27]. Among the reported mutations in ALSoD, the cysteine (*Cys*) residue in the 6th position reported mutations including *Cys6Trp*, *Cys6Phe*, *Cys6Ser*, and *Cys6Gly* which are associated with individuals with fALS [28] (https://alsod.ac.uk/output/gene.php/SOD1).

 We aim to investigate the effects of missense mutations in the SOD1 protein at the 6th *Cysteine* residue in patients with fALS. Although there have been clinical reports on these mutations, there is still ambiguity surrounding their specific impact on the structure of the SOD1 protein and the resulting molecular mechanisms. The objective of this study is to provide insights into the impact of these mutations on the conformation and functionality of the SOD1 protein, thereby contributing to our understanding of their role in fALS.

## Materials and methods

### SOD1 mutation retrieval and pathogenicity prediction

The mutations *Cys6Ser*, *Cys6Trp*, *Cys6Phe*, and *Cys6Gly* were selected from the ALSoD. The PROVEAN (Protein Variation Effect Analyzer) tool [29], PolyPhen-2 (Polymorphism Phenotyping v2) [30], Meta-SNP, and PhD-SNP were used to evaluate the impact of missense mutations on the function of the SOD1 at the sequence level. The three aforementioned tools were utilised via the PhD-SNP server [31].

### Evaluating structural stability of SOD1 mutants

The DUET server was utilised to analyse the effect of missense mutations on the structural stability. DUET integrates the mCSM and SDM methodologies to generate a consensus prediction. It achieves this by employing support vector machines to merge their outcomes into an enhanced predictor [32]. The DynaMut2 server, which assesses the effect of missense mutations on protein dynamics and stability by analysing changes in vibrational entropy, was employed to predict the impact of missense mutations on overall stability [33]. PremPS web server was used to analyse the alterations in intramolecular interaction resulting from the missense mutations. PremPS quantifies the alterations in Gibbs free energy resulting from individual mutations in protein stability and presents a visual depiction of the modifications in bonding arrangement [34]. The three-dimensional structure of SOD1 (PDB ID: 1PU0) was used as a reference structure for the above-mentioned analysis.

### Molecular dynamic (MD) studies

The wild SOD1 three-dimensional structure (1PU0) was validated using ProSA-Web and PROCHECK servers. The mutants were generated using the Chimera software’s rotamer function and minimised for further analysis. The GROMACS v2021.1 software [35] was used to run 100 ns molecular dynamics (MD) simulations on wild-type and mutant forms of SOD1 (*Cys6Trp*, *Cys6Phe*, *Cys6Ser*, and *Cys6Gly*). The initial topology files were generated using the pdb2gmx module and the OPLS-AA (optimised potential for liquid simulations) force field. The protein was solvated using the TIP3P water model in a cubic box measuring 1 nm on each side. The system was then equilibrated and subsequently neutralised by the addition of Na^+^ (sodium) and Cl^−^ (chloride) ions. The boundary effect was eliminated using the periodic boundary condition (PBC) applied, and the protein was energy minimised using the steepest descent algorithm. Before production runs, the system was set up with NVT (constant number of particles volume and temperature) at 300 K and NPT (constant number of particles pressure and temperature) at 1 atm pressure for 500 ps each to ensure the pre-equilibration of the system at constant temperature and pressure. MD simulation was performed on the system for 100 ns, recording trajectory data at intervals of 100 ps. This data was subsequently used to analyse the root mean square deviation (RMSD), radius of gyration (Rg), root mean square fluctuation (RMSF), solvent-accessible surface area (SASA), and hydrogen bonding patterns of the wild-type and mutant proteins.

### Assessing the impact of mutations on SOD1 dimerisation

The binding free energy between the individual monomeric subunits of *Cys6WT* and mutants (*Cys6Trp, Cys6Phe, Cys6Gly*, and* Cys6Ser)* were determined using gmx_MMPBSA v1.52 plugin GROMACS. The analysis employs the Molecular Mechanics/Poisson-Boltzmann Surface Area (MM/PBSA) method for the calculation of total binding energy [36] using the MD trajectory. The entire MD trajectories obtained for the *Cys6WT* and mutants’ dimers were used for the calculation, and the following scheme was employed for the calculation of the binding free energy.

$$\Delta G_{\mathrm{binding}}=G_{\mathrm{dimer}}-\left(\mathrm G\cdot_{\mathrm{Monomer}1}+\mathrm G\cdot_{\mathrm{Monomer}2}\right)$$

Further, the MD trajectories of the wild (*Cys6WT)* and mutant (*Cys6Trp, Cys6Phe, Cys6Gly*, and* Cys6Ser)* dimers were analysed for their intermolecular hydrogen bond occupancy at the dimer interface using the gmx hbond plugin.

## Results and discussion

### Prediction pathogenicity of SOD1 mutants

The SOD1 mutations at the 6th position (*Cysteine*) were retrieved from the ALSoD specific to fALS. The functional impact of the missense mutations was analysed using PROVEAN, PolyPhen-2, Meta-SNP, and PhD-SNP, and the results are tabulated in Table 1.

**Table 1:** Functional impact of SOD1 missense mutations predicted using PROVEAN, PolyPhen-2, PhD-SNP and Meta-SNP

Mutants	PROVEAN		PolyPhen-2		PhD-SNP		Meta- SNP	
	Score	Interpretation	Score	Interpretation	Score	Interpretation	Score	Interpretation
*Cys6Gly*	− 9.492	Deleterious	1.0	Probably damaging	0.803	Disease-causing	0.673	Disease causing
*Cys6Phe*	− 8.793		1.0		0.807		0.652	
*Cys6Ser*	− 7.878		1.0		0.694		0.554	
*Cys6Trp*	− 8.818		1.0		0.839		0.730	

Table 1 indicates that all mutations occurring at the 6th position of the SOD1 protein were determined to be deleterious, with an increased likelihood of causing disease. Based on PROVEAN analysis, a score lower than − 2.5 will result in a harmful effect [29], and all four mutations are predicted to have a significantly negative impact. Similarly, the PolyPhen-2 tool predicts the impact of amino acid substitution in proteins based on structural, functional, and evolutionary information [30], and the tool predicts all the substitutions are probably damaging. The SVM vector-based PhD-SNP classifier developed using protein sequences and the functional profiles [31] embedded in the Meta-SNP server collectively predicted all the mutations to be disease-causing. Our analysis revealed that all the SOD1 mutants had a detrimental effect on biological function, indicating their pathogenic nature.

### Effect of missense mutations on protein stability

**Fig. 1:**
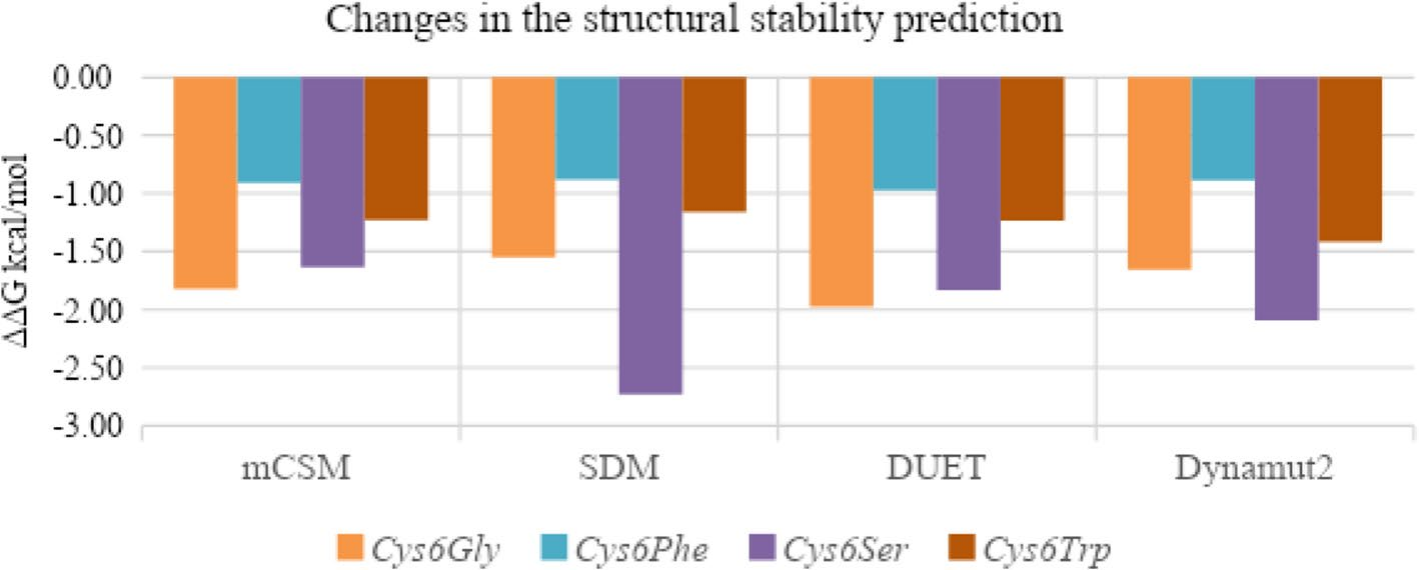
Analysis of stability changes in SOD1 due to mutations, as predicted by mCSM, SDM, DUET, and Dynamut2

The changes in the protein structure due to mutations are analysed using mCSM, SDM, DUET, and Dynamut2 servers. The results are predicted as changes in the Gibbs free energy (ΔΔG), where a negative value corresponds to the destabilising effect and a positive value corresponds to stabilising effect [32]. All four mutations in SOD1 showed a destabilising effect (Fig. 1), while *Cys6Ser* and *Cys6Gly* were found to be highly destabilising.

**Fig. 2:**
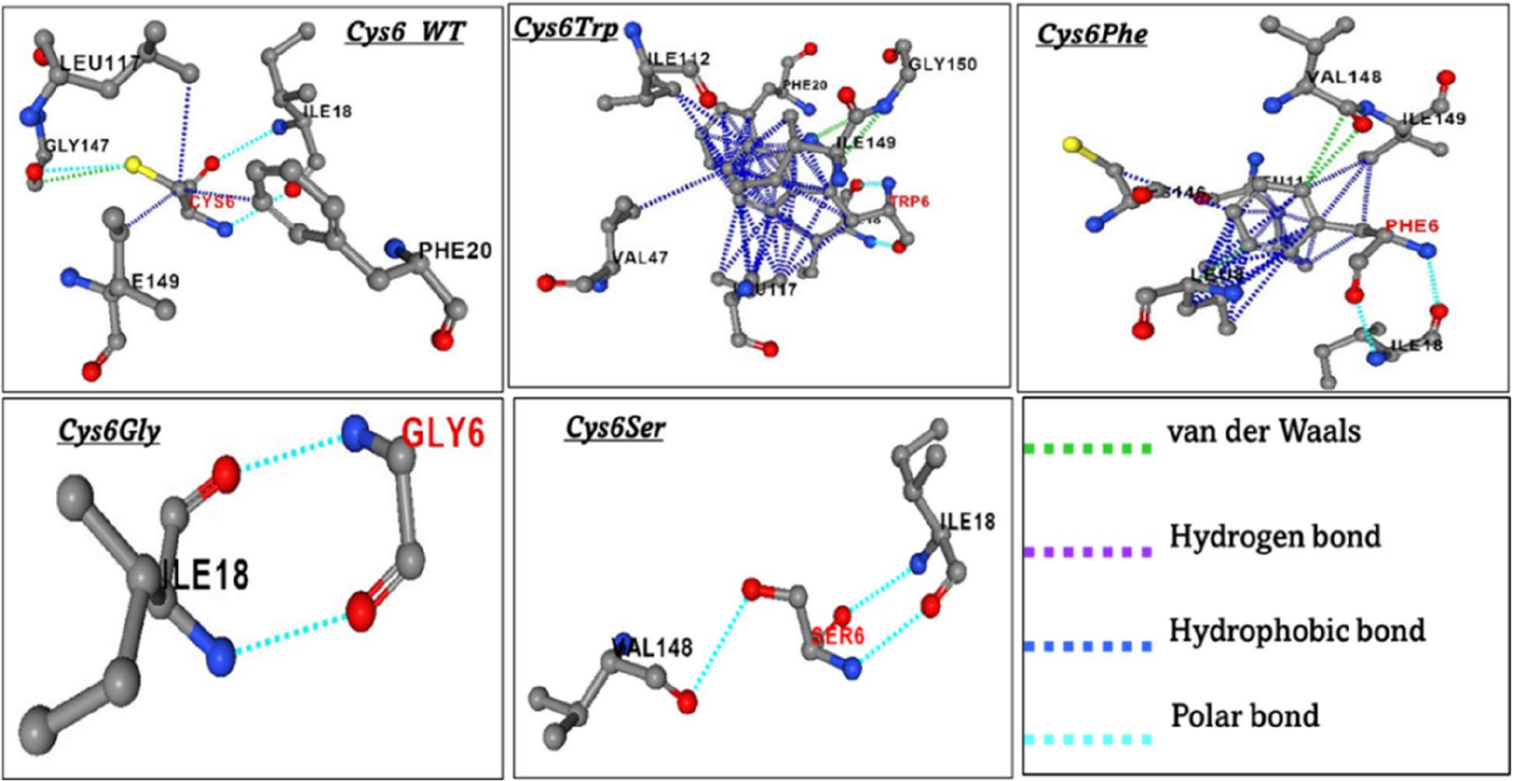
Changes in the intramolecular interactions of SOD1 protein due to mutations

As the mutations showed a destabilising effect, the impact of the mutations on the intramolecular binding pattern was deciphered using PremPS, and the results are represented in Fig. 2.

Our analysis showed no changes in the polar interactions between *Cys6* and *Ile18* due to mutations, and additionally, the *Cys6Ser* mutant showed polar bonds with *Val148*. The hydrophobic interaction in the SOD1 protein was found to be altered in the *Cys6Trp* and *Cys6Phe* mutations compared to the *Cys6WT* (Fig. 2). Interestingly, the mutations *Cys6Ser* and *Cys6Gly* resulted in complete loss of hydrophobic interactions. Furthermore, in the wild type, only *Gly147* formed van der Waals (VDW) interactions. In the *Cys6Trp* mutation, the amino acid residues *Leu117*, *Ile112*, *Ile149*, and *Gly150* formed intramolecular VDW interactions, while in the *Cys6Phe* mutation, the residues *Val148* and *Leu8* did. The VDW interactions were completely lost in the case of mutations *Cys6Ser* and *Cys6Gly*. The intramolecular hydrogen bonding was found to be similar in wild-type and mutant *Cys6Trp*, where *Ile18* formed the hydrogen bond, whereas this was completely lost in the case of *Cys6Ser* and *Cys6Gly* mutants*.* The summary of changes in bonding patterns between the amino acid residues due to mutations is provided in Table 2.

**Table 2:** Changes in intramolecular interactions in SOD1 protein due to mutations

Interaction	*Cys6 WT*	*Cys6Trp*	*Cys6Phe*	*Cys6Ser*	*Cys6Gly*
Polar	Leu117, Ile18	Ile18	Ile18	Val148, Ile18	1le18
Hydrophobic	Ile149, Phe20, Leu117	Ile18, Val47, Ile112, Leu117, Phe20, Ile149	Ile149, Leu117, Leu8, Cys146		
VDW	Gly147	Leu117, Ile112, Ile149, Gly150	Val148, Leu8	-	
H bond	Ile18	Ile18	-	-	

Key: *VDW* van der Waals interactions.

### Molecular dynamic studies for structural stability

**Fig. 3:**
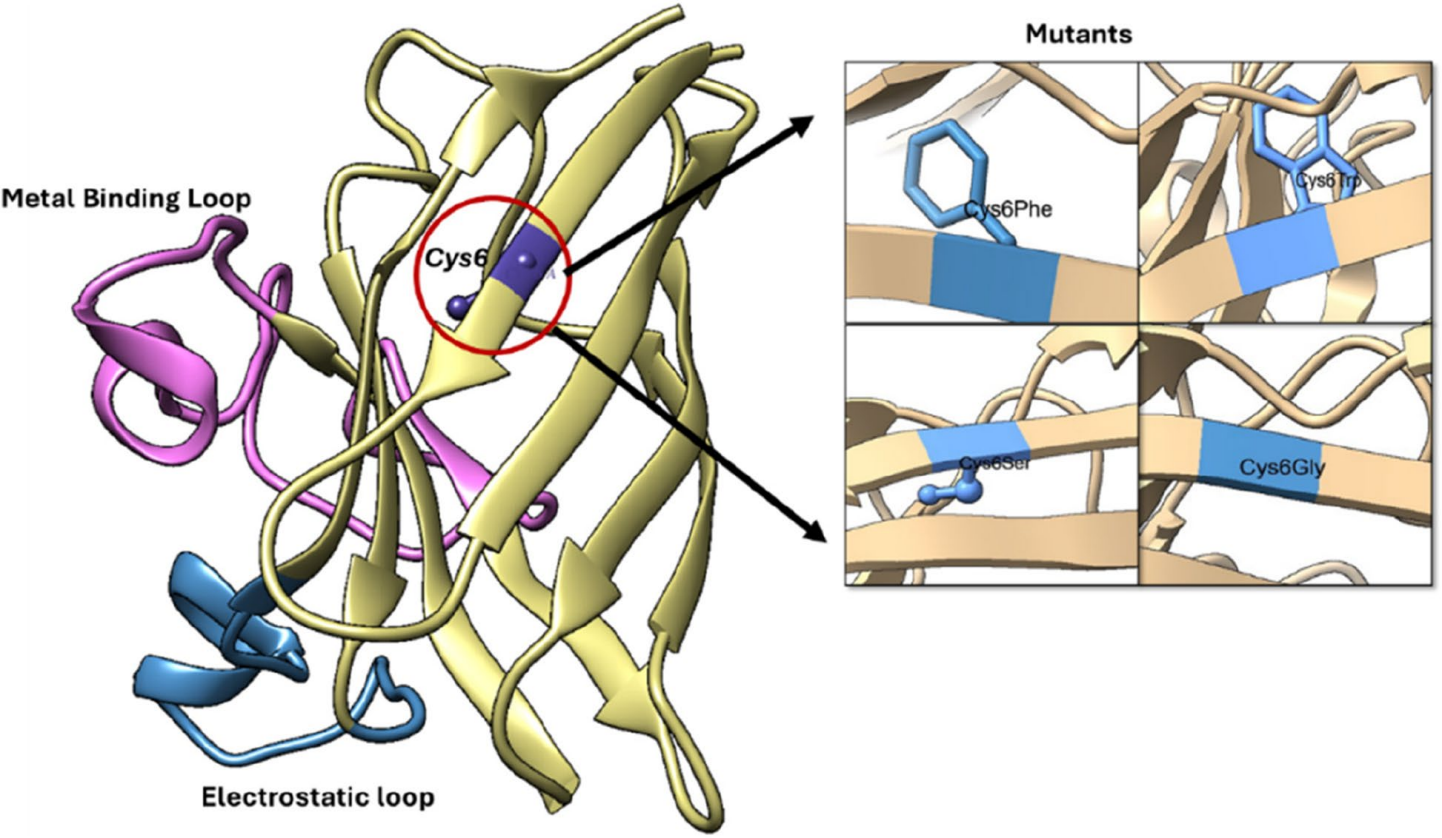
** a** Schematic representation of three-dimensional structure of *Cys6WT* with metal binding loop, electrostatic loop, and *Cys6* highlighted. **b** Mutations at the 6th *Cysteine* residue of SOD1.

The three-dimensional structure of SOD1 was downloaded, and the mutants were modelled using the Rotamer function embedded in Chimera software. The SOD1 WT (*Cys6WT*) and mutant structures are shown in Fig. 3.

To understand the impact of SOD1 mutations (*Cys6Trp*, *Cys6Phe*, *Cys6Ser*, and *Cys6Gly*) on the protein structure, MD simulations were performed for a duration of 100 ns. For the time-dependent function of molecular dynamic simulations, the trajectories were used to analyse changes in RMSD, RMSF, Rg, and SASA.

**Fig. 4:**
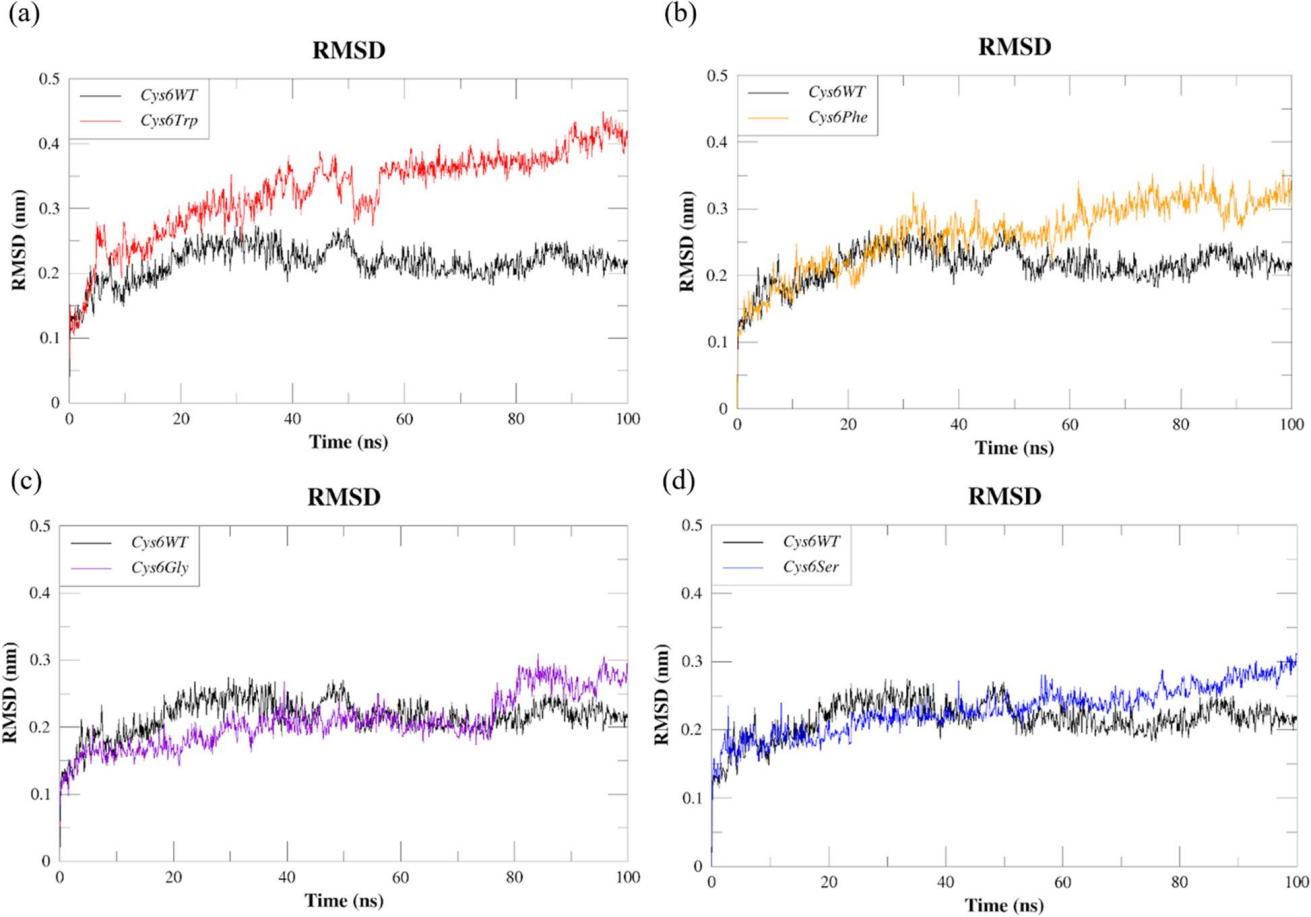
Comparative RMSD plots of SOD1 wild type (*Cys6WT*) and mutants: **a** *Cys6Trp*, **b** *Cys6Phe*, **c** *Cys6Gly*, and **d** *Cys6Ser*

To evaluate how the mutation affects the stability of the SOD1 protein structure, the RMSD values for the backbone residues of both the wild-type and mutant proteins were calculated for all atoms in the starting structure, and the results are shown in Fig. 4a–d. The occurrence of higher RMSD values is indicative of structural variations from the original state, potentially resulting in decreased stability and impaired protein functionality [37]. As shown in Fig. 4a, the *Cys6WT* protein showed early equilibration with a RMSD value of 0.2 nm, while the mutant *Cys6Trp* showed initially a higher RMSD of 0.3 nm, further after 50 ns equilibrated with a value of 0.4 nm indicating the destabilising effect of mutation on the overall structure of SOD1. Similarly, the mutant *Cys6Phe* initially had a lower RMSD of 0.2 nm, but after 50 ns, the protein remained equilibrated with a higher RMSD of 0.3 nm, as shown in Fig. 4b. Interestingly, the mutations *Cys6Gly* and *Cys6Ser* showed an overall stabilising effect on the SOD1 structure with RMSD values similar to those of *Cys6Trp* with minor fluctuations (Fig. 4c and d). With a RMSD similar to *Cys6WT*, *Cys6Gly* showed the least destabilising effect on the SOD1.

Based on our research, it was observed that the mutations *Cyc6Gly* and *Cycs6Ser* exhibited minimal RMSD fluctuations in comparison to *Cys6WT*. This indicates that there were no significant alterations in stability. In contrast, the mutants *Cys6Trp* and *Cys6Phe* exhibited greater RMSD fluctuations compared to the native wild-type SOD1. It is evident that these mutations (*Cys6Trp* and *Cys6Phe*) significantly impacted the structural stability under normal physiological conditions.

**Fig. 5:**
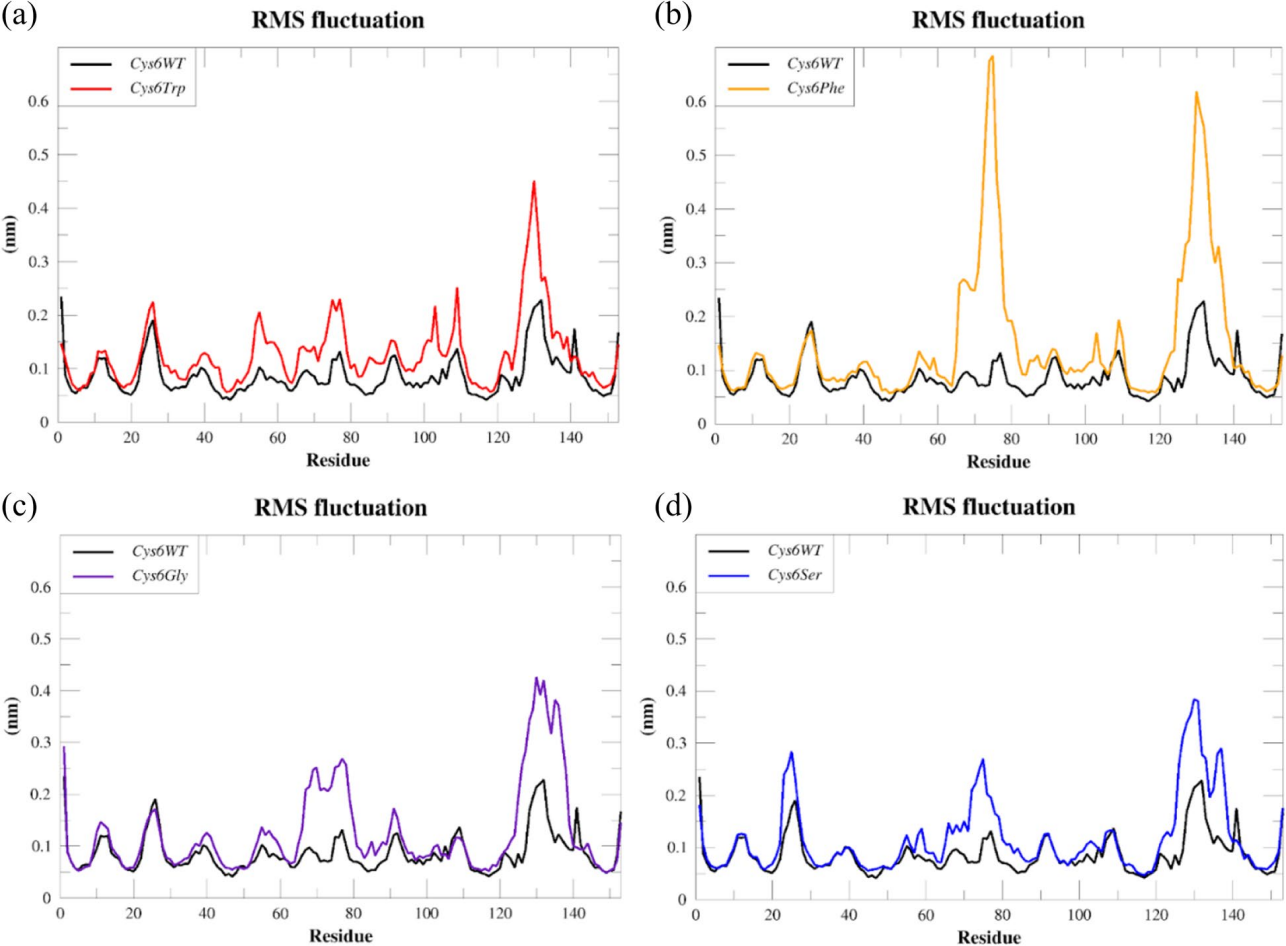
Comparative RMSF plots of the Cα atoms residues of SOD1 wild type (*Cys6WT*) and mutants: **a** *Cys6Trp*, **b** *Cys6Phe*, **c** *Cys6Gly*, and **d** *Cys6Ser*

The RMSF analysis was performed on the MD trajectories to calculate the fluctuations at the Cα atoms of individual amino acid residues in the wild type and mutants, and the results are represented in Fig. 5. Comparative analysis of the RMSF values of *Cys6Trp* and *Cys6WT* showed fluctuations up to 0.2 nm in most parts of the protein. The electrostatic loop (region 122–143), on the other hand, showed peak variations of 0.45 nm Fig. 5a, indicating the diminished catalytic activity of the protein. Although the mutation was observed at the N-terminal end, the amino acid fluctuation pattern in this part was found to be similar to *Cys6WT*.

In the case of the *Cys6Phe* mutant, disulphide sub loop (regions 55–61) and the metal binding sub loop (regions 62–83) showed the highest fluctuations of 0.65 nm in comparison with the *Cys6WT*, which showed a value as low as 0.1 nm in the same region. In a similar way, the electrostatic loop (regions 122–143) also showed fluctuations up to 0.6 nm in comparison with the *Cys6WT*, shown in Fig. 5b. The *Cys6Phe* mutant demonstrated the largest variations of 0.65 nm in the disulphide sub loop (regions 55–61) and the metal binding sub loop (regions 62–83), while the *Cys6WT* showed a much lower value of 0.1 nm in the same region. Similarly, the electrostatic loop (regions 122–143) exhibited fluctuations up to 0.6 nm in comparison to the *Cys6WT*, as illustrated in Fig. 5b.

The RMSF analysis of the *Cys6Gly* mutant revealed variations of 0.3 nm in the metal binding loop and 0.4 nm in the electrostatic loop, as compared to the *Cys6WT*. Further, the remaining portion of the mutant protein exhibited a comparable pattern of fluctuation to that of *Cys6WT*. Figure 5c displays the comparative analysis of the RMSF patterns of *Cys6WT* and *Cys6Gly*. The RMSF pattern of the mutant *Cys6Ser* showed similar fluctuations observed among *Cys6Gly* (Fig. 5d). In addition, the *Cys6Ser* also showed fluctuations from the 20th to the 25th regions of the protein.

Overall, the RMSF analysis revealed the impairment of the functioning of the SOD1 mutants due to the changes in the metal binding loop and the electrostatic loop, which are crucial for the functioning of the protein. Among the reported *Cys6* mutations, the *Cys6Phe, Cys6Gly*, was reported among the Japanese and Chinese patients of fALS, leading to severe clinical symptoms and rapid disease progression [38–41]. The fluctuations in the metal binding and electrostatic loop identified through our study provide insights into the diminished function of SOD1, linking the clinical symptoms and the structural alteration due to mutations *Cys6Phe* and *Cys6Gly*. Meanwhile, the mutation *Cys6Trp* was associated with reduced SOD1 activity and mild disease symptoms [41], this can be associated majorly with the alteration in the electrostatic loop which is involved in the catalytic activity of SOD1. In contrast, the mutations *Cys6Ser* showed lesser fluctuation in the catalytic and the metal binding loop providing the confirmation to the reports by Brotherton et al. [42], where *Cys6Ser* was reported among fALS patients found to be stabilising the SOD1.

**Fig. 6:**
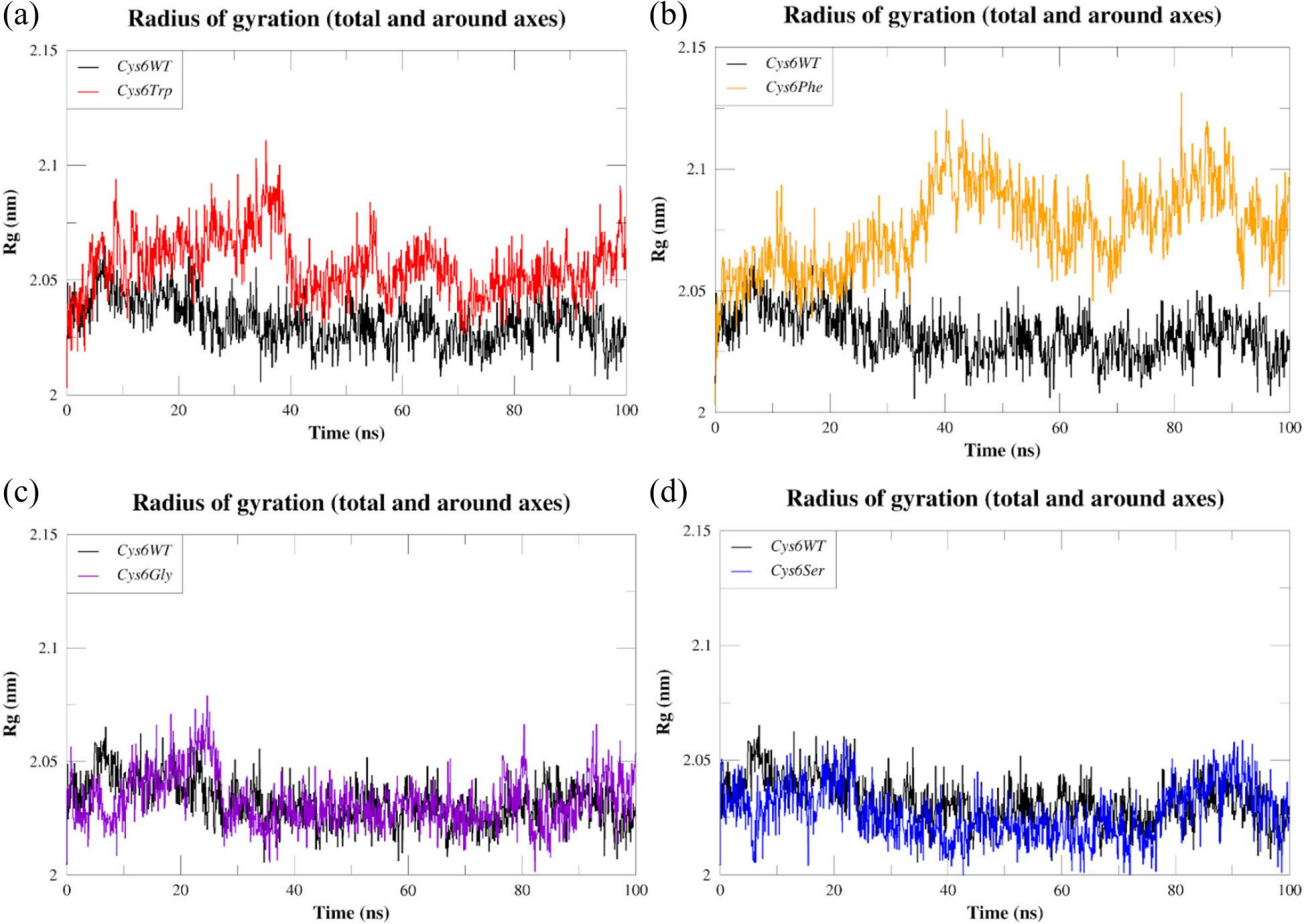
Comparative Rg plots of SOD1 wild type (*Cys6WT*) and mutants: **a** *Cys6Trp*, **b** *Cys6Phe*, **c** *Cys6Gly*, and **d** *Cys6Ser*

The radius of gyration (Rg) is a crucial measure for evaluating the compactness and overall stability of protein structures. Variations in Rg can indicate conformational changes that may impact protein functionality. A decrease in Rg generally signifies a more compact and stable conformation, while an increase suggests unfolding or destabilisation events [43]. The Rg values from the trajectories were used to decipher the compactness of SOD1 upon mutations, and the results are shown in Fig. 6a–d. The analysis revealed changes in compactness in the cases of *Cys6Trp* and *Cys6Phe*, with average Rg values of 2.05 nm and 2.07 nm, respectively, in contrast to the average of 2.03 nm of *Cys6WT*. The mutants *Cys6Gly* and *Cys6Ser* showed no changes in compactness, with similar Rg values to those of *Cys6WT*.

Our research indicates that the mutant *Cys6Phe* had greater Rg values, suggesting a difference in compactness compared to *Cys6WT*. Similarly, the *Cys6Trp* variant exhibited alterations in compactness. However, *Cys6Gly* and *Cys6Ser* did not exhibit any alterations in conformational flexibility or rigidity.

**Fig. 7:**
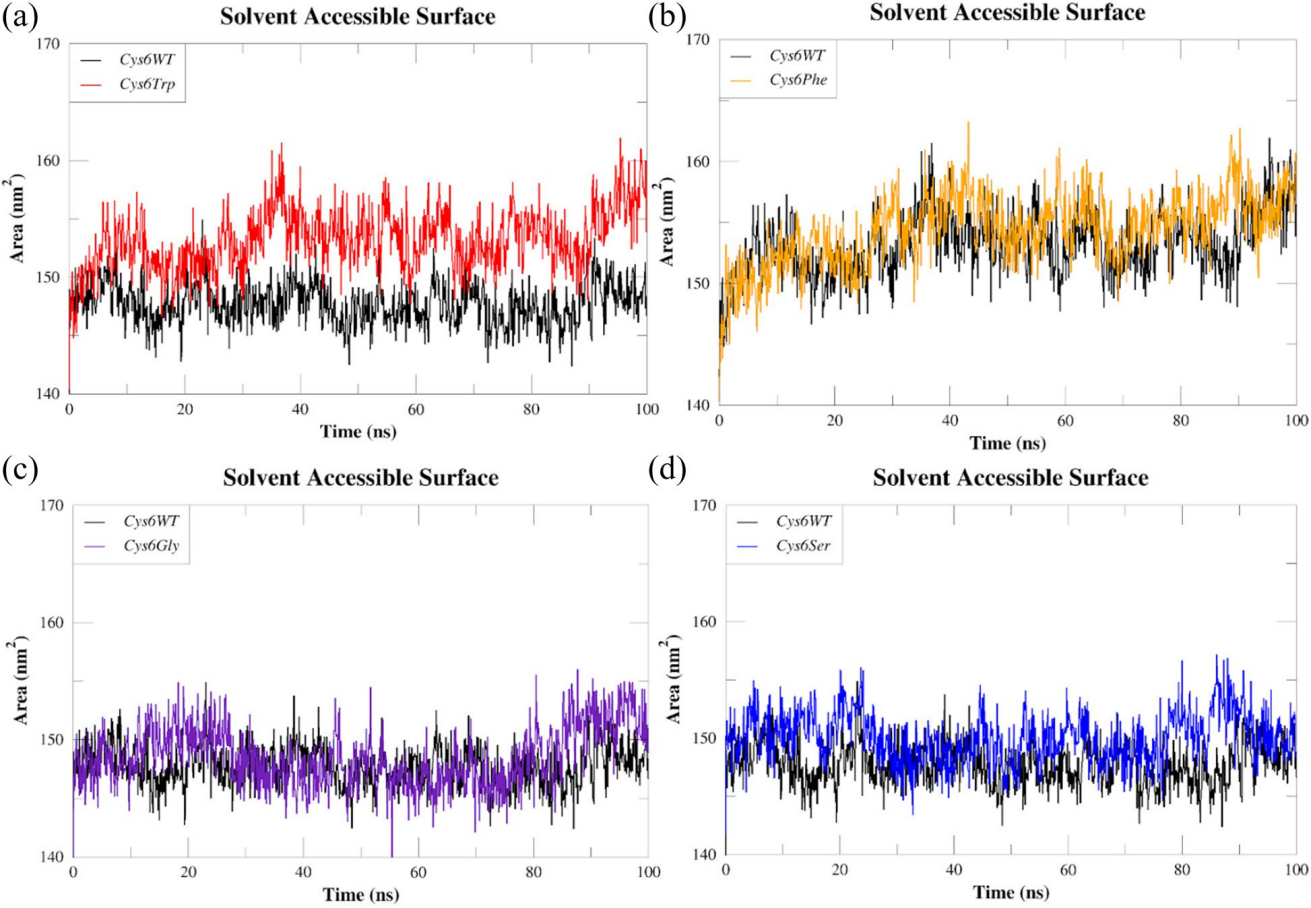
Comparative SASA plots of SOD1 wild type (*Cys6WT*) and mutants: **a** *Cys6Trp*, **b** *Cys6Phe*, **c** *Cys6Gly*, and **d** *Cys6Ser*

The SASA analysis studies of mutants showed increased solvent accessibility among *Cys6Trp* and *Cys6Phe* with an average value of 153.19 nm^2^ and 154.42 nm^2^, respectively. The *Cys6WT* showed an average of 147.72 nm^2^. The *Cys6Gly* and *Cys6Ser* showed an average SASA value of 148.5 nm^2^ and 150.08 nm^2^ which were slightly higher than the *Cys6WT*. The comparative SASA values obtained for the *Cys6WT* and mutant are shown in Fig. 7a–d.

Consistent with our Rg analysis results, the SASA study demonstrated that *Cys6Trp* and *Cys6Phe* exhibit more flexibility, leading to an expansion of the solvent-accessible surface area as a consequence of protein misfolding.

**Fig. 8:**
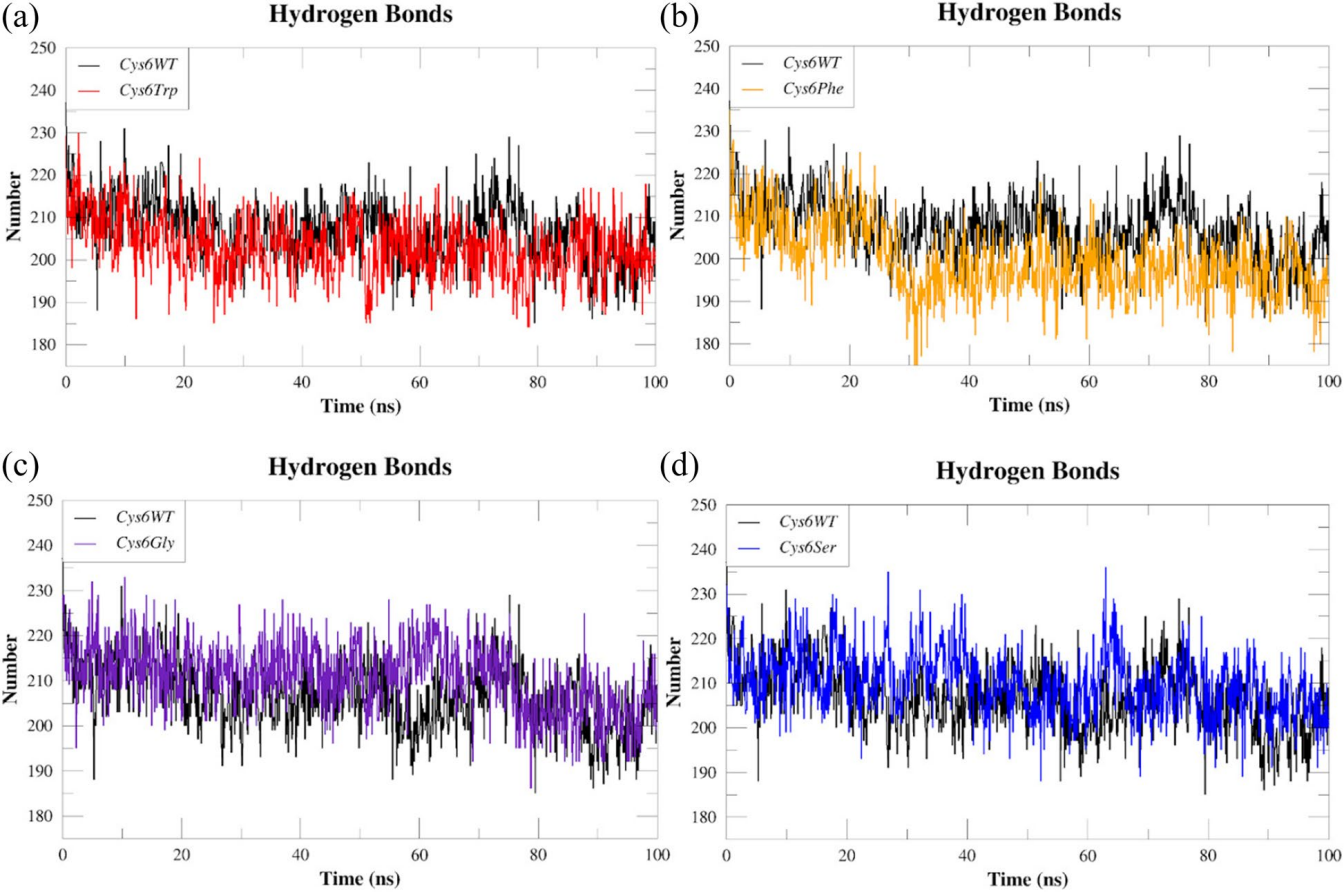
Comparative hydrogen bonding pattern analysis of SOD1 wild type (*Cys6WT*) and mutants **a** *Cys6Trp*, **b** *Cys6Phe*, **c** *Cys6Gly*, and **d** *Cys6Ser*

The MD trajectory data was used to investigate the stability of SOD1 wild-type and mutants, which is conferred by the intra-hydrogen bonds. The MD trajectory data was used to study the stability of the SOD1 wild type and mutants, which is conferred by the intra-hydrogen bond. Figure 8a–d shows the intra-hydrogen bonding seen in the paths of both SOD1 mutants and its wild-type form. The average number of intra-hydrogen bonds for *Cys6Trp* was 202, whereas it was 199 for *Cys6Phe*, 210 for *Cys6Gly*, and 209 for *Cys6Ser*. In contrast, *Cys6WT* showed an average of 206 hydrogen bonds.

Overall, our analysis demonstrated a slight increase in the numbers of intra-hydrogen bonds in *Cys6Gly* and *Cys6Ser*, while *Cys6Trp* and *Cys6Phe* showed a marginal decrease.

#### Assessing the impact of mutations on SOD1 dimerisation

The dimerisation stability of SOD1 due to mutations was studied by analysing the changes in total binding free energy changes at the interface of monomers. The MM/PBSA analysis of the SOD1 dimer revealed variations in binding energy associated with different mutations at the *Cys6* position. The overall changes in the binding free energy at the interface of the SOD1 dimer are represented in Table 3.

**Table 3:** Changes in the binding free energy at the dimer interface of SOD1 monomer, calculated by MM/PBSA

	***Cys6WT***	***Cys6Trp***	***Cys6Phe***	***Cys6Gly***	***Cys6Ser***
Total binding energy (kcal/mol)	− 57.96	− 62.64	− 47.11	− 52.96	− 50.91
SD	6.47	6.56	3.97	5.43	4.85
SEM	0.2	0.2	0.13	0.17	0.15

**Fig. 9:**
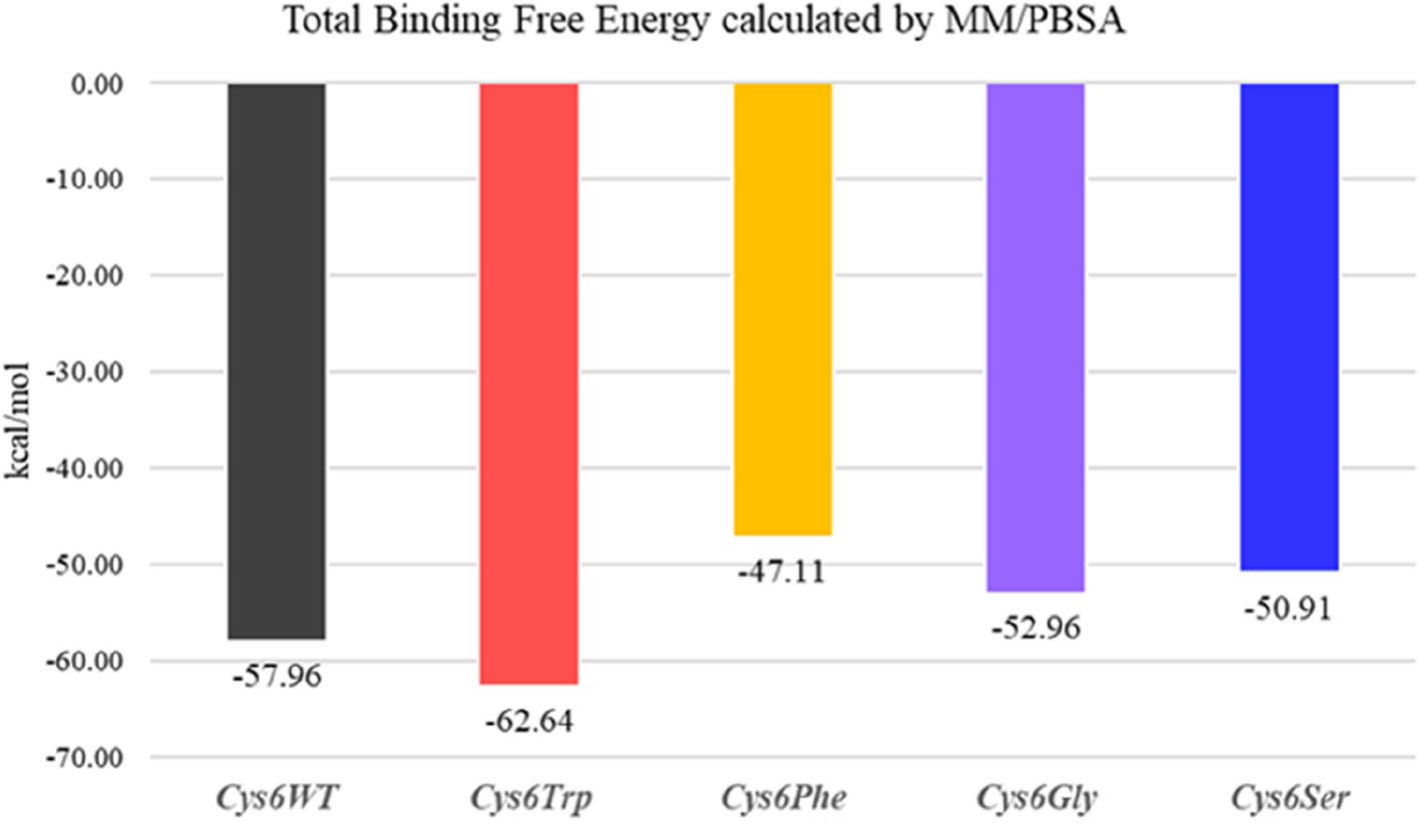
Comparative binding free energy of *Cys6WT* and *Cys6Trp*, *Cys6Phe*, *Cys6Gly*, and *Cys6Ser* calculated using MM/PBSA

The *Cys6WT* (wild type) SOD1 showed a total binding energy of − 57.96 kcal/mol, indicating stable dimerisation. Among the mutants, *Cys6Trp* showed the most favourable binding with a total energy of − 62.64 kcal/mol, representing a 7% increase in binding free energy compared to the wild type. On the other hand, *Cys6Phe* showed the least favourable binding energy (− 47.11 kcal/mol), corresponding to an 18.63% decrease in comparison with *Cys6WT*. *Cys6Gly* and *Cys6Ser* exhibited intermediate binding energies of − 52.96 kcal/mol and − 50.91 kcal/mol, reflecting decreases of 8.53% and 12.1%, respectively, in comparison to the wild-type *Cys6WT*. The overall results obtained for the total binding free energy for the wild type and mutants are shown in Fig. 9.

The amino acid residue *Cys6* is located at the dimerisation interface of the SOD1 protein, where it plays a crucial role in the dimerisation process [44]. Our findings suggest that substituting *Cys6* with bulkier or hydrophobic residues, *Cys6Trp*, enhances the stability of the dimer interface. The increased stability observed with the *Cys6Trp* mutation can be attributed to hydrophobic interactions and π-π stacking, which are well-known stabilising factors in protein interactions [45]. This trend aligns with previous reports, such as the study by Eleutherio et al. (2021), which highlighted the importance of hydrophobic contacts at the dimer interface for maintaining SOD1 stability. Our results reinforce this finding, particularly with the *Cys6Trp* mutation [9]. In contrast, the substitutions of *Cys6* with other residues, *Cys6Phe, Cys6Gly*, and *Cys6Ser*, lead to reduced binding affinity leading to dimer destability. The reduced binding energy at the dimer interface due to the *Cys6Phe* mutation could alter dimer stabilisation, potentially increasing the risk of aggregation.

**Fig. 10:**
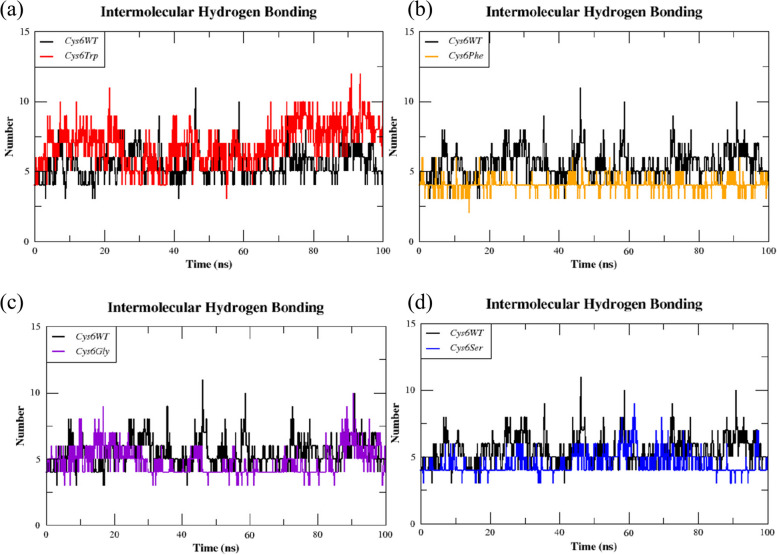
The hydrogen bond occupancy between the subunits WT SOD1 and mutant dimers: **a** *Cys6Trp*, **b** *Cys6Phe*, **c** *Cys6Gly*, and **d** *Cys6Ser*

The hydrogen bonding interactions at the dimer interface of SOD1 were analysed over a time period of 100 ns using GROMACS trajectory data and the results are represented in Fig. 10.

The *Cys6WT* showed an average of 5.4 hydrogen bonds, whereas the mutants *Cys6Trp, Cys6Phe, Cys6Gly*, and* Cys6Ser *had 7.0, 4.0, 4.8, and 4.5 hydrogen bonds respectively. Hydrogen bonding interactions at the dimer interface are critical for the structural stability and functional integrity of SOD1. Reports have suggested that the hydrogen bonds at the dimer interface play a significant role in stabilising the subunits of SOD1, contributing to the overall stability of the protein [2]. Our analysis provides insights on the relative contributions of specific residues to the stability of the SOD1 dimer interface, with the mutant *Cys6Trp* exhibiting the highest hydrogen bonding strength and while *Cys6Phe* showed the least stabilising effect.

Overall, our study reveals that the *Cys6* residue plays a crucial role in the dimerisation stability of SOD1, with substitutions impacting binding energy and hydrogen bonding interactions. The *Cys6Trp* mutation showed the most favourable binding energy and increased stability due to enhanced hydrophobic interactions and π-π stacking, aligning with previous findings. In contrast, the *Cys6Phe* mutation significantly reduced binding affinity, showing the least stabilising effect on the dimer interface. The decrease in binding energy observed in *Cys6Phe*, along with reduced hydrogen bonding interactions, suggests that this mutation destabilises the dimer, potentially increasing the risk of protein aggregation. These findings underscore the importance of *Cys6* in maintaining SOD1’s structural integrity and function.

## Conclusion

The occurrence and progression of ALS are influenced by the mutational landscape of SOD1. Recent research has identified over 185 of these mutations, which play a role in the development and progression of the disease. In this study, we investigated the pathogenic potential and stability changes resulting from mutations Cysteine 6 residue of SOD1. We employed various prediction tools to analyse changes in the bonding patterns and assess the overall structural impact through MD simulations. Our study has provided an in-depth understanding of the changes brought about by the mutations (*Cys6Trp,* *Cys6Phe,* *Cys6Ser,* and *Cys6Gly*) on the functionality of SOD1. In addition, molecular dynamics (MD) simulations thoroughly evaluated the overall effect on the protein structural dynamics and dimerisation. The findings presented in this study provide valuable insights into the molecular mechanisms that drive structural changes caused by mutations. These insights contribute to a deeper understanding of the implications of these changes for pathogenicity. Through extensive computational analysis, we have uncovered the intricate ways in which these mutations cause substantial changes in the SOD1’s catalytic activity and contribute to the understating of the SOD1 dimerisation. The findings obtained from this study enhance our understanding of the molecular mechanisms through which these mutations affect the function of SOD1. These findings not only deepen our understanding of SOD1’s role in biological processes but also open doors for future investigations focused on developing targeted interventions to alleviate the effects of these mutations.

## Data Availability

No datasets were generated or analysed during the current study.
